# Assessing outcomes of large-scale public health interventions in the absence of baseline data using a mixture of Cox and binomial regressions

**DOI:** 10.1186/1471-2288-14-2

**Published:** 2014-01-07

**Authors:** Thierry Duchesne, Belkacem Abdous, Catherine M Lowndes, Michel Alary

**Affiliations:** 1 Département de mathématiques et de statistique, Université Laval, 1045 avenue de la Médecine, Québec, Québec, QC G1V 0A6, Canada; 2 Axe Santé des populations et pratiques optimales en santé, Centre de recherche du CHU de Québec, Québec, Québec, Canada; 3 Département de Médecine sociale et préventive, Université Laval, 1050 avenue de la Médecine, Québec, Québec, QC G1V 0A6, Canada; 4 Health Protection Agency, London, UK

**Keywords:** Avahan, Censoring, Change-point, Consistent condom use, Female sex workers, Generalized estimating equations, Length bias, Logistic regression, Mixture model, Time-dependent covariate

## Abstract

**Background:**

Large-scale public health interventions with rapid scale-up are increasingly being implemented worldwide. Such implementation allows for a large target population to be reached in a short period of time. But when the time comes to investigate the effectiveness of these interventions, the rapid scale-up creates several methodological challenges, such as the lack of baseline data and the absence of control groups. One example of such an intervention is Avahan, the India HIV/AIDS initiative of the Bill & Melinda Gates Foundation. One question of interest is the effect of Avahan on condom use by female sex workers with their clients. By retrospectively reconstructing condom use and sex work history from survey data, it is possible to estimate how condom use rates evolve over time. However formal inference about how this rate changes at a given point in calendar time remains challenging.

**Methods:**

We propose a new statistical procedure based on a mixture of binomial regression and Cox regression. We compare this new method to an existing approach based on generalized estimating equations through simulations and application to Indian data.

**Results:**

Both methods are unbiased, but the proposed method is more powerful than the existing method, especially when initial condom use is high. When applied to the Indian data, the new method mostly agrees with the existing method, but seems to have corrected some implausible results of the latter in a few districts. We also show how the new method can be used to analyze the data of all districts combined.

**Conclusions:**

The use of both methods can be recommended for exploratory data analysis. However for formal statistical inference, the new method has better power.

## Background

Assessing the impact of large-scale interventions is essential in the context of scarce resources [1]. For instance, Ng et al. [2] investigate impact of Avahan, the HIV/AIDS initiative of the Bill & Melinda Gates Foundation in India [3,4], on HIV prevalence in pregnant women and they cite examples of assessments of other large-scale interventions [5-7]. Due to ethical concerns and other constraints, scale-up is often rapid and simultaneous, so there is no randomization or control group [8]. Furthermore, in populations targeted by such large-scale programs it is often the case that no extensive or quality baseline data on population health prior to the start of the intervention are available. To add to the difficulty, it is often not possible to have longitudinal or prospective follow-up for a period that is long enough to provide sufficient data in these target populations, especially in the field of HIV: either the incidence of disease is too low to allow sufficient statistical power if the general population is studied or the level of loss to follow-up is high when studying high-risk populations.

Nevertheless, the impact of these programs must be assessed and the assessment of intermediate outcomes is essential as a prerequisite for understanding the mechanism through which the interventions may have an impact. One strategy proposed by Lowndes and collaborators [9] consists of a retrospective construction of the baseline information using survey data obtained during the course of the Avahan intervention. Specifically they asked female sex workers (FSWs) if they consistently use condoms with all of their occasional clients and if so, since when. They also asked the FSWs when they started their career as sex worker. They showed how this approach enabled them to simply, yet accurately, reconstruct time trends of consistent condom use (CCU) in the target population. Testing for a difference in CCU pre- and post-intervention from such reconstructed data, however, proved to be more difficult. An approach based on binomial regression with a linear link function and generalized estimating equations (GEE) yielded results that were somewhat questionable for districts where CCU was already high in the pre-intervention period, even suggesting that prevention may have had a significant negative impact on CCU in these districts.

Our objective in this paper is to propose an alternative to the GEE-based method to make inferences about pre- and post-intervention differences on the basis of such reconstructed data. Though we apply the method to the analysis of condom use in FSWs, the proposed method is a combination of standard analysis tools and is applicable to any context where one seeks to make inferences about the effect of an intervention on the rate of occurrence of a given outcome when baseline data are not available but can be assessed retrospectively using a questionnaire.

In terms of broad statistical strategy, the idea is to use the fact that the data observed are times-to-events and that the goal of the analysis is to make inferences about a change-point in the rate of occurrence of one of these events. Recently, Minard and collaborators [10] compared an approach based on logistic regression with GEE to a Markov modeling approach to test whether an intervention program had a significant effect on smoking cessation behavior. They observed that the GEE approach lacked power to detect time-covariate interactions. As has been noticed in other contexts [11,12], this suggests that methods based on direct time-to-event modeling tend to make more efficient use of the information contained in this type of dataset and should be at the core of any efficient methodological approach. The Markov model proposed by Minard et al. cannot, however, be used in our context. Indeed, their model was built under the assumption of stationarity, i.e., that the intensities of transition from one state (e.g., smoker) to another (e.g., non-smoker) are constant in time. Because our primary interest is to make inferences about a change in a rate of occurrence after a specified date, stationarity is obviously not an option that we can consider. An additional difficulty that we will see is that in the FSW data, an important proportion of the individuals have an event time exactly equal to zero (i.e., they consistently used the condom at the beginning of their sex work career). This implies that the model to be used must have a probability mass at time zero, along the lines of the zero-inflated Cox model used by Grouwels and Braekers [13] in their study of ethanol-induced anesthesia. Our context is simpler than theirs, however, because we do not have to deal with left-censored observations, which enables us to fit their model with standard binomial and Cox regression software.

The paper is organized as follows. In the methods section we describe the type of datasets under consideration, quickly review the GEE method, describe our proposed Cox-binomial approach and give the details of the simulation study and real data application through which the methods will be compared. The results of these comparisons are summarized in the results section. A discussion of the strengths and weaknesses of the proposal along with ideas for further research are outlined in the discussion section. An appendix with technical details as well as a file with the R code required to implement the new method are also provided.

## Methods

### Study design

The method that we propose can be applied to cross-sectional studies where participants are randomly recruited in a specified target population at a specific point τ in calendar time (or over a relatively short period of time about τ). For instance in the condom use study, women involved in sex work at well-defined locations were sampled in 2006 (with the exception of a few districts where the sampling was done in 2007 or 2008). Even though we do not consider it in this paper, survey weights can presumably be added to the methods to handle data obtained with sampling schemes other than simple random sampling. Each participant recruited is asked to provide two dates: (i) the date at which she became part of the target population and (ii) the date at which some permanent status change took place. In the condom use study, each woman was asked (i) at what date she started her career as a sex worker and (ii) if she consistently uses the condom with her occasional clients and if so, since when. The objective is to make inferences about differences in how permanent status change takes place before and after a given calendar time, say *t*_0_, on the basis of such data. In the condom use study, we are interested in the detection of changes in how FSWs start CCU before and after 1-1-2004, date at which the Avahan program started its prevention intervention [3]. Because of the high level of heterogeneity of the HIV epidemic in India [14] the nature of the intervention varies from district to district and the evaluation was planned at the district-level [4,15]. Consequently, separate inferences for each district are desired. But separate inferences are not usually the norm; we also perform a unified analysis. To avoid repetition, in the sequel we present the methods in terms of the condom use study, even though they are applicable in a broader context.

### Inference based on generalized estimating equations

Getting crude estimates of the rate of CCU at any given calendar time *t*, say *R*_*t*_ = *U*_*t*_/*N*_*t*_, is fairly straightforward with the data that are available. As illustrated in Table one of Lowndes et al., the denominator *N*_*t*_ of the rate is the number of women in the survey who begun sex work before time *t* while the numerator *U*_*t*_ is the number, among the *N*_*t*_ women who begun sex work before *t*, who started CCU before *t*. As long as the intervention does not have an impact on the length of the career as FSW (more on this in the Discussion), then these crude estimates of the rate of condom use should be unbiased.

Under the assumption that *R*_*t*_ is steadily increasing with *t*, the question of interest is whether this rate of increase changes after calendar time *t*_0_. A first visual assessment can be done by plotting *R*_*t*_ as a function of *t* for several values of *t* and seeing whether the slope of the relationship changes after *t*_0_. Lowndes et al. formally tested whether the difference in the average slope was different before and after 1-1-2004 by fitting a binomial regression model with linear link, with a subject being a FSW, the repeated response being whether the FSW consistently uses the condom in a given calendar year and the covariates being indicators corresponding to the calendar years and testing the appropriate contrast for significance.

There are two main drawbacks with this GEE-based method. First, each FSW is not observed at every given time *t* at which the rate *R*_*t*_ is calculated. Hence treating the condom use statuses at each *t* as a cluster of longitudinal observations may not be appropriate. But most importantly, the method is very likely to conclude that the slope after time *t*_0_ is not different from, or maybe even significantly lower than, the slope before time *t*_0_ when the rates before *t*_0_ are already high. This is easily seen with an example: if the condom use rate goes from 70% to 90% from 2001 to 2003, then it would have to go from 90% to more than 110% from 2004 to 2006 in order for the average slope to be steeper after 1-1-2004 than before 1-1-2004, which is of course impossible. Hence this test of change in average slope cannot possibly assess whether CCU increases after a given calendar time if the rate of CCU is already high before that calendar time. Moreover, as discussed previously, the literature suggests that an approach based on time-to-event modeling is likely to be more powerful.

### Inference based on a mixture Cox model with time-dependent covariate

Our objective is to propose a method based on time-to-event modeling that, unlike the GEE approach, (i) does not lose its efficiency when condom use is already high before *t*_0_ and that (ii) only uses the two dates provided by the FSWs (time at start of career as FSW and time at start of CCU) as data inputs.

#### Assumption on career as female sex worker

**Figure 1:**
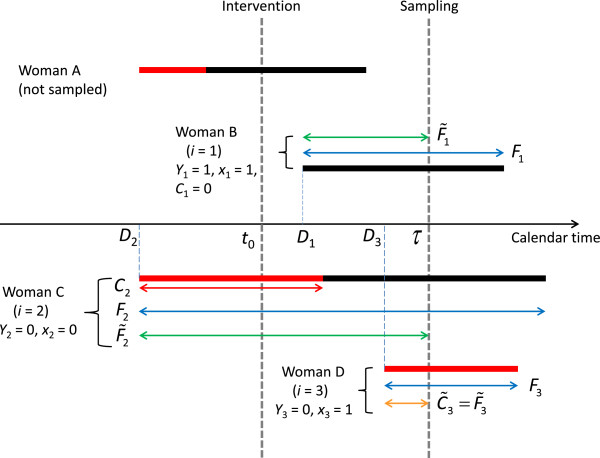
**Illustration of the notation.** Thick segments: four careers as FSW, with the portion of career without condom use in red and the portion of career with condom use in black. The lengths of the double arrows are the values of the variables $C_{i}$, ${\tilde{C}}_{i}$, $F_{i}$, ${\tilde{F}}_{i}$, when applicable. Vertical dashed lines are drawn at the time of sampling (*τ*) and at the time of the intervention ($t_{0}$).

Our strategy is based on the following assumption in terms of the chronology of CCU. We suppose that when they begin their career, FSWs either immediately start CCU, either they do not. If they do not, then they might eventually start CCU at some time before the end of their career, or they might not. Mathematically, let *D*_*i*_ denote the calendar time at which woman *i* begins her career as FSW. Let *Y*_*i*_ = 1 if she consistently uses the condom when she starts working as a FSW and let *Y*_*i*_ = 0 otherwise. Let *C*_*i*_ denote the amount of time during which woman *i* works as a FSW before starting CCU. Note that when *Y*_*i*_ = 1 woman *i* consistently uses the condom at the start of her career and thus *C*_*i*_ = 0. On the other hand, when *Y*_*i*_ = 0 woman *i* does not use the condom as soon as she starts her career and therefore *C*_*i*_ > 0. If we denote by *F*_*i*_ the length of the career of woman *i* as a FSW, then if *C*_*i*_ < *F*_*i*_, woman *i* will start to consistently use the condom before the end of her career. Conversely, if *C*_*i*_ > *F*_*i*_, then woman *i* will not use the condom consistently during her career. This notation is illustrated in Figure 1.

#### Observed values of the variables

Given the study design (see Figure 1), woman *i* may only be included in the study if she started her career before τ (*D*_*i*_ < τ) and if her career does not end before τ (*D*_*i*_ + *F*_*i*_ > τ); this is the case for women B, C and D in Figure 1. Clearly, we can deduce the values of *D*_*i*_ and *Y*_*i*_ from the information provided by the FSWs taking part in the study. However we only get a lower bound on *F*_*i*_ (all we know is that *F*_*i*_ > τ – *D*_*i*_) and therefore its value is right-censored for every FSW in the study, i.e., 100% of the observations of *F*_*i*_ are right-censored. As for *C*_*i*_, we know its exact value if FSW *i* has started CCU before τ (women B and C in Figure 1), but we only know that it is greater than τ – *D*_*i*_ (right-censored) if she does not use the condom consistently at the time of data collection τ (woman D in Figure 1).

#### Model and method

Our main interest is in the distribution of *C*_*i*_. We will suppose that the *C*_*i*_ of different FSWs are independent. Because of the probability mass at *C*_*i*_ = 0 (FSWs who started CCU at the beginning of their career), we use a mixture model [13]. We suppose that *C*_*i*_ = 0 (i.e., that *Y*_*i*_ = 1) with probability *π*_*i*_ and that *C*_*i*_ > 0 (i.e., that *Y*_*i*_ = 0) with probability 1-*π*_*i*_. Then for the conditional distribution of *C*_*i*_ given *C*_*i*_ > 0, we can use any standard survival model.

Because the objective of the study is to assess changes in CCU after a given calendar time *t*_0_, we must model *π*_*i*_ and the distribution of *C*_*i*_ when *C*_*i*_ > 0 as functions of *t*_0_; of course, other covariates can be included in the model and we give the details of the general model in Appendix A. There are several modeling options for *π*_*i*_. In our case, because we are mainly interested in comparing how FSWs start CCU before and after the intervention, we use a simple binomial regression model. Let *x*_*i*_ = 0 if FSW *i* started her career before *t*_0_ (e.g., woman C in Figure 1) and *x*_*i*_ = 1 if FSW *i* started her career after *t*_0_ (e.g., women B and D in Figure 1). Then we can make inferences about changes in *π*_*i*_ after *t*_0_ by performing the binomial regression with *Y*_*i*_ as outcome and *x*_*i*_ as covariate:

(1) $$g\left(\pi_{i}\right)=\beta_{0}+\beta_{1}x_{i}$$

where *g*() is a known link function. The most common link is the logit function, but if the event to be modeled has high prevalence, then using a log link in Eq. 1 will yield regression coefficients that can be interpreted as log prevalence ratios, which is usually preferable to log odds ratios. Other links such as the probit or complementary log-log can also be used instead of the logit link. If desired, the effect of time can be modeled in a more complex fashion by changing the right-hand side of Eq. 1, for example by changing the step function with a cut point at *t*_0_ to a broken line with a cut point at *t*_0_. If other covariates are available, they can also be included on the RHS of Eq. 1.

As for the distribution of *C*_*i*_ when *C*_*i*_ > 0, define the time-dependent covariate *z*_*i*_(*t*) that takes on value 0 when *t* < *t*_0_ and value 1 when *t* ≥ *t*_0_; once again, other covariates (or stratification variables) can be used and a more general formulation of the model is given in the Appendix. Then a change in the risk of starting to consistently use the condom at time *t*_0_ can be estimated by fitting a Cox model to the dataset comprised of FSWs for whom *C*_*i*_ > 0, with *C*_*i*_ as response and *z*_*i*_(*t*) as time-dependent covariate:

(2) $$h\left(t\right)=h_{o}\left(t\right)\exp\left\{\gamma z_{i}\left(t\right)\right\}$$

Under the assumption (which we discuss below) that *C*_*i*_ and *F*_*i*_ are independent, we show in Appendix A that the two models can be fitted independently of each other with standard binomial and Cox regression software. Effect of *t*_0_ on each model can be assessed separately, or the likelihood ratio (or Wald) statistics to test whether *x*_*i*_ and *z*_*i*_(*t*) are significant in their respective models (i.e., *β*_1_ = 0 in Eq. 1 and *γ* = 0 in Eq. 2) can be added together. Under the null hypothesis of no change in CCU at *t*_0_, this sum of two likelihood ratio (or Wald) statistics approximately follows a chi-squared distribution with two degrees-of-freedom.

The Cox-binomial method described above can also be used on clustered data. As we show in the Appendix, all one has to do is fit the two models (Cox and binomial) with methods that will yield robust “sandwich” variance estimates of the parameter estimators and then use a Wald test on two degrees of freedom instead of a likelihood ratio test.

As a referee suggested, this Cox-binomial model can be approximated by a single Cox model by imputing a very small positive time to the observations that have the event at time zero. This modeling approach is easier to implement and can be more easily generalized, as is the case when one wishes to include random effects, for instance. However we do not advocate the use of this approximation when the individuals who have an event time equal to zero represent a non-negligible proportion of the sample, as will be the case in our data application.

### Comparison of the methods by simulation

We ran some simulations to compare the properties of the two methods. The simulation algorithm and the parameter values were chosen so that the simulated samples were roughly similar to the samples in the real dataset to be discussed below. In all runs, we simulated a population of 800 women. Their dates of birth were randomly generated according to a normal distribution with mean 1973 and standard deviation 7. The age at which they began sex work was simulated from a normal distribution with mean 22 and standard deviation 4. The duration of their career as FSWs (*F*_*i*_) followed a Weibull distribution with shape 3 and scale 40. Condom use was simulated according to the Cox-binomial model, whereby each woman started to consistently use the condom at the beginning of her career with probability *π*_1_ if she started sex work before 1-1-2004 (time *t*_0_) and with probability *π*_2_ if she started sex work after 1-1-2004. For women who did not consistently use the condom at the beginning of their career, we simulated the duration of sex work until CCU (*C*_*i*_) from a piecewise exponential distribution with rate *h*_1_ before 1-1-2004 and rate *h*_2_ after 1-1-2004. The specific sets of values of *π*_1_, *π*_2_, *h*_1_ and *h*_2_ used in the simulations are given in Tables 1 and 2. We set 1-1-2009 as the time of data collection (time τ), which generated samples of between 350 and 450 FSWs contributing to the estimation of the binomial regression parameters and to the GEE method, and between 70 and 290 FSWs contributing to the estimation of the Cox model parameter, which is quite comparable to the sample sizes observed in the real data analyses reported in Table 3. Tests of the hypothesis of no change in CCU after 1-1-2004 were carried at the 5% significance level.

**Table 1:** Values of the parameters associated with condom use in the simulation study

**Consistent**	**Probability of condom**	**Rate of condom**
**condom use**	**use at the beginning**	**acquisition during**
	**of career**	**career**
Low	*π* = 0.2	*h* = 0.05
Medium	*π* = 0.5	*h* = 0.10
High	*π* = 0.8	*h* = 0.25

**Table 2:** **Proportion of the 1**,**000 simulated samples for which the null hypothesis of no change in consistent condom use was rejected at the 0.05 level**

**Consistent condom use**		**Proportion of samples leading to rejection of *H***_**0**_		
**Pre 1-1-2004**	**Post 1-1-2004**	**GEE-linear**	**GEE-logit**	**Cox-binomial**
**Low**	**Low**	0.067^*^	**0.059**	**0.059**
Low	Medium	0.883	0.916	0.998
Low	High	1.000	1.000	1.000
**Medium**	**Medium**	**0.056**	0.068^*^	**0.062**
Medium	High	0.734	0.984	1.000
**High**	**High**	**0.056**	**NA**	**0.050**

Rows with writing in boldface represent settings where the null hypothesis is true. Starred values (*) in the rows in boldface indicate rejection rates significantly (at the 5% level) different from 0.05.

**Table 3:** **Application of the Cox**-**binomial and GEE methods to the data on condom use by FSWs with their occasional clients for 21 districts in India**

	**Cox**-**binomial model**									**GEE method**	
**District**	***n***_**Cox**_	${\hat{h}}_{1}$	${\hat{h}}_{2}$	***p***_**Cox**_	***n***_**logit**_	${\hat{\pi }}_{1}$	${\hat{\pi }}_{2}$	***p***_**logit**_	***p***_**Total**_	**%Diff**	***p *** **<0.05**
Belgaum	199	0.069	0.295	<0.001	397	0.366	0.775	<0.001	<0.001	−0.5	NO
Bellary	234	0.090	0.274	<0.001	398	0.183	0.760	<0.001	<0.001	−2.7	NO
Chennai	265	0.073	0.446	<0.001	349	0.143	0.413	<0.001	<0.001	13.6	YES
Chitoor	360	0.012	0.107	<0.001	395	0.026	0.230	<0.001	<0.001	7.7	YES
Coimbatore	306	0.006	0.144	<0.001	325	0.020	0.117	<0.001	<0.001	12.2	YES
Dharmapuri	306	0.020	0.262	<0.001	387	0.049	0.657	<0.001	<0.001	16.5	YES
East Godavari	303	0.067	0.314	<0.001	392	0.149	0.518	<0.001	<0.001	4.5	NO
Guntur	324	0.012	0.345	<0.001	386	0.068	0.532	<0.001	<0.001	21.9	YES
Madurai	269	0.044	0.263	<0.001	319	0.097	0.304	<0.001	<0.001	12.1	YES
Mumbai BB	156	0.069	0.112	0.578	379	0.576	0.629	0.369	0.573	−0.9	NO
Mumbai NBB	144	0.064	0.072	0.983	354	0.557	0.711	0.011	0.041	−0.8	NO
Mysore	328	0.031	0.191	<0.001	420	0.120	0.377	<0.001	<0.001	8.7	YES
Prakasam	374	0.003	0.123	<0.001	402	0.026	0.204	<0.001	<0.001	10.1	YES
Pune BB	74	0.202	0.261	0.884	399	0.769	0.942	<0.001	<0.001	−3.9	YES
Pune NBB	60	0.112	0.130	0.054	251	0.689	0.870	<0.001	<0.001	0.0	NO
Salem	249	0.035	0.313	<0.001	319	0.106	0.364	<0.001	<0.001	13.9	YES
Shimoga	192	0.059	0.225	<0.001	338	0.158	0.641	<0.001	<0.001	2.9	NO
Thane BB	54	0.337	0.500	0.752	397	0.847	0.913	0.082	0.209	−5.6	YES
Thane NBB	64	0.241	0.311	0.662	377	0.735	0.894	<0.001	<0.001	−3.5	NO
Visakhapatnam	350	0.042	0.386	<0.001	405	0.043	0.500	<0.001	<0.001	18.8	YES
Yevatmal	57	0.131	0.435	<0.001	148	0.328	0.800	<0.001	<0.001	0.6	NO

TOTAL 4668 7140.

Columns 2 and 6: Number of FSWs contributing the Cox (*n*_Cox_) and binomial (*n*_logit_) regressions. Columns 3 and 4: mean rate of condom acquisition during career before 1-1-2004 (${\hat{h}}_{1}$) and after 1-1-2004 (${\hat{h}}_{2}$). Columns 7 and 8: Mean probability of condom use at beginning of career before 1-1-2004 (${\hat{\pi }}_{1}$) and after 1-1-2004 (${\hat{\pi }}_{2}$). Columns 5, 9 and 10: *p*-values of the likelihood-ratio test of no difference pre- and post-intervention in condom acquisition (*p*_Cox_), condom use at beginning of career (*p*_logit_) and combined tests (*p*_Total_). Columns 11 and 12: Estimate of the difference between the average yearly slopes of consistent condom use before and after 1-1-2004 with the GEE approach with a *p*-value for the test that this difference is significantly different from 0. “BB” stands for “brothel-based” and “NBB” stands for “Non brothel-based”.

## Results

### Simulation study

As can be seen from Table 2, when condom use does not change in time, both methods yield fairly unbiased inferences, with rejection levels that are never significantly different from 0.05 with the Cox-binomial approach, and only once barely superior to the rejection limit of 0.064 for each of GEE with linear and logit links. Unfortunately, when initial condom use was high, the GEE method with logit link failed to converge. The power of both methods is very good when an increase in CCU after 1-1-2004 follows a low CCU before 1-1-2004. However when CCU before 1-1-2004 is medium, the GEE method loses some of its power to detect an increase in CCU after 1-1-2004, most especially with the linear link. On the other hand, the Cox-binomial approach is able to maintain its very high power even when initial CCU is medium.

### Application to the Indian data

#### GEE method with linear link

We reanalyzed without sampling weights (for comparability with the Cox-binomial method; results remain very similar to those published in [9]) the data on occasional clients collected from integrated behavioural and biological assessment (IBBA) [16,17] considered by Lowndes et al. and give the results in Table 3. The overall sample size for the 21 districts was 7140 FSWs who all contributed to the binomial regression model and the GEE analysis. Of these FSWs, 4668 did not start CCU at the beginning of their career and contributed to the Cox model analysis. A gross summary of the results of Lowndes et al. is that the estimated proportion of CCU in FSWs increased from 2001 to 2006. However the estimated average yearly rate of increase was lower after 1-1-2004 than before that date for five of the 21 districts considered, (significantly so for two of these districts, Pune brothel-based and Thane brothel-based), suggesting a negative effect of the intervention in these districts where the proportion of CCU was already high; this is barely plausible.

#### Re-analysis with the Cox-binomial method

Results of the analysis of the same data with the Cox-binomial approach are summarized in Table 3. Although we fitted the semi-parametric Cox model described in Eq. 2 that did not assume piecewise constant hazards, we reported the mean condom acquisition rate pre- and post-intervention in each district as ${\hat{h}}_{1}$ and ${\hat{h}}_{2}$, respectively, in Table 3 for descriptive purposes. Though the findings with the Cox-binomial by and large agree with those obtained with the GEE method, there are a few interesting differences. First, all estimates obtained with the Cox-binomial approach suggest an increase in CCU (be it CCU at the start of the career as FSW or acquisition of CCU during the course of the career as FSW for those who did not consistently use the condom at the beginning of their career) after 1-1-2004. Moreover, except for Mumbai brothel-based and Thane brothel-based, all of these increases are statistically significant at the 5% level. In all instances, the p-value of the combined and binomial models lead to the same conclusion, while for a few districts the p-value of the Cox model is not significant when the p-value of the combined model is. The sample sizes in the two districts in which the binomial regressions are not significant are not the ones with smaller samples. There are 6 districts for which the p-value associated to the coefficient of the Cox model is not significant, and for 4 of these 6 districts, the sample size is small. As could be expected from theoretical arguments and the results of the simulation study, differences between the results obtained with the two approaches happen in districts where CCU is already high before 1-1-2004 (i.e., ${\hat{h}}_{1}$ and ${\hat{\pi }}_{1}$ high).

We also tried the single Cox model approximation by fitting the Cox model above to the data from all FSWs where the event time for FSWs starting CCU at career start set equal to 1 day. Since we have many districts where initial CCU is high, the method leads to questionable results in some districts. The full results are reported in Additional file 1 available on the journal’s website.

#### Analysis with the Cox-binomial model combining all districts and with additional covariates

To have a population-averaged effect of the intervention, we combined the data from all the districts and re-did the Cox-binomial analysis. For the binomial part of the model, we used GEE with an independence working assumption and a logit link; attempts with a log link failed to converge. For the Cox part, we used the same model as for the district-wise analyses, but fitted the model using the marginal approach (see [18], chapter 8). We first performed this combined analysis with the same covariates as the district-wise analyses, then repeated it with the addition of the year at which the FSW started sex work in the binomial model and calendar year as a time-varying covariate in the Cox model. The results obtained are summarized in Table 4. For these data, 7,140 FSWs contributed to the estimation of the prevalence of condom use at the beginning of sex work, with estimates of 0.279 before the intervention and 0.586 after. Not surprisingly, in the Cox-binomial model with no other covariate this difference is highly significant (p-value < 0.0001). However when the year of beginning of sex work is entered in the model, the effect of the intervention ceases to be significant. As for incidence of CCU acquisition during sex work, 4,668 contributed to the inferences and we observed a rate of 0.049 before the intervention and 0.236 after. Again, this difference is highly significant in the Cox part of the Cox-binomial analysis without any other covariate (p-value < 0.0001). With calendar year as a time-varying covariate in the model, the effect of the intervention remains positive and significant with a p-value of 0.0026. When we compute the Wald statistic described in the appendix we obtain a value of 11.41, which yields a p-value of 0.0033, and thus we reject the null hypothesis that the intervention has no effect.

**Table 4:** **Application of the Cox**-**binomial approach for clustered data described in the appendix to the Indian data combining all 21 districts**

**Model with intervention effect only**	**Estimate**	**Robust s.e**.	**p**-**value**
**Binomial part**			
Intervention	1.297	0.23	<0.0001
**Cox part**			
Intervention	1.496	0.16	<0.0001
**Model with intervention and year**			
**Binomial part**			
Intervention	−0.255	0.17	0.13
Year	0.444	0.09	<0.0001
**Cox part**			
Intervention	0.723	0.24	0.0026
Year	0.298	0.07	<0.0001

## Discussion

### Summary

We proposed a new method to test whether a significant change occurred at a given point in calendar time *t*_0_ in how individuals in a target population experience a permanent status change, motivated by a study of how the acquisition of CCU by FSWs in India changed after the beginning of an intervention that began on 1-1-2004. The new method is based on a Cox model with a time-dependent covariate to which a probability mass at time zero is added; standard binomial regression is used to model this probability mass. We showed how this new approach can easily be implemented with standard software that fit the binomial regression and Cox models and how it can be extended to deal with clustered data. We contrasted the new method with an existing approach that compares the average slope of the status change rate before and after *t*_0_ using GEE. We found that inferences based on the two methods were quite comparable when the prevalence of status change was low before *t*_0_, but that the new Cox-binomial approach was more powerful to detect changes when prevalence of status change before *t*_0_ increased. When we applied the new method to the data on CCU in FSWs in India analyzed by Lowndes et al., we obtained that CCU after 1-1-2004 increased in all 21 districts considered, significantly so in all but two of them. In contrast, with the GEE approach only 10 significant increases were found and five decreases were observed, two of the latter being significant decreases. When applied simultaneously to the data from all 21 districts and when correcting for calendar time, the method suggests that increase in CCU at career start is mostly associated with calendar time while acquisition of CCU in the course of the career as FSW is positively associated to both the intervention and calendar time.

### Strengths

The method proposed in this paper has several strengths. First and foremost, it remains powerful at detecting change even when the prevalence of permanent status change is high before *t*_0_, which is the main drawback of the GEE method that motivated this work. Not only did this gain in power stand out in the simulation study, but also in the application of the method to the CCU in FSWs study. Second, its application only requires the two dates measured in the data (date at which the individual enters the target population and date at which status change takes place) as data inputs, unlike the GEE method which creates a cluster of observations of arbitrary size for each individual. Third, its implementation in practice is quite simple and can be done using standard software for Cox and binomial regression. In this paper, all analyses were performed with R [19] (and the R code to implement the method is available in “Additional file 2), but they could as easily have been performed with SAS [20] or Stata [21]. Finally, the method is flexible. In this paper we only considered a binomial regression and a Cox model. But the basic strategy remains valid if one uses different models for the probability masses at zero and the survival times. Furthermore, the method can easily be adapted to accommodate clustered data.

### Limitations

There are some limitations to the method of which the users should be aware. An important technical issue is that all inferences in the GEE method or in the method proposed in this paper rely on an assumption that cannot be tested with the type of dataset described in this study. Indeed, the validity of the inference relies on the independence between the amount of time spent in the target population (random variable *F*_*i*_) and the amount of time spent in the target population before status change takes place (random variable *C*_*i*_). If there is no such independence, then inferences on the distribution of *C*_*i*_ cannot be made independently of *F*_*i*_ and both the proposed Cox-binomial method and the GEE approach break down. This may be further compounded by the fact that the distribution of *F*_*i*_ observed in the sample is not the same as the distribution of *F*_*i*_ in the target population due to the length bias phenomenon inherent to any prevalent cohort sampling scheme [22], whereby individuals with longer prevalent periods are more likely to be part of a cross-sectional sample.

Another weakness of the method proposed (that is also a weakness of the GEE approach) is that it assumes that changes in the probability of status change is instantaneous at time *t*_0_, while in reality the probability may change smoothly between *t*_0_ and some other time *t*_1_ > *t*_0_, or may be instantaneous but delayed at some other time *t*_1_ > *t*_0_. Furthermore *t*_1_ may be unknown.

It can also be argued that the scope of the simulation study presented here is somewhat limited, as we did not fit models where the model assumptions were false. But because our objective was to determine whether the Cox-binomial approach overcomes the loss of power of the GEE method when the model assumptions are true, we feel that additional simulations under more general conditions are not necessary at this stage and should be considered in future research.

A key limitation that arises when we apply our method to the study of CCU in FSW in India is that the method that we propose here does not address the potential bias due to the “before-after” design of this kind of study. In this particular case, it is very difficult to control for many sources of bias that could cause CCU to evolve in time because no district that was not part of the intervention could be added to the analysis.

### Future research

The problem of inference from length biased data has been studied in the statistical literature, for example using nonparametric methods [22] or inverse weighting [23]. However the existing methods mainly consider univariate distributions and data arising from prevalent cohort studies with follow-up. It would be interesting to see what minimal additional assumptions and/or data would be required to derive estimation methods for the bivariate distribution of *C*_*i*_ and *F*_*i*_, or at the very least to derive a test of independence between these two variables. The fact that the distribution of *C*_*i*_ has a point mass at zero and a hazard rate that may both have a change point at calendar time *t*_0_ represents an additional modeling challenge.

As a matter of fact it should be possible to model the manner in which the point mass or the hazard change following the intervention at time *t*_0_ in a more flexible way. For instance in the case where we suppose the change to take place at an unknown time *t*_0_, fitting the proposed Cox-binomial model with several values of *t*_0_ and keeping the value of *t*_0_ yielding the largest likelihood ratio test statistic is a sensible avenue. However in this case the *p*-value of the tests cannot be approximated by simple chi-squared distributions anymore. Determination of such *p*-values has been studied for an ordinary Cox model [24], but further work is needed for the Cox-binomial mixture.

## Conclusion

Our aim in this paper was to improve the efficiency of the methods used to assess intervention outcomes on the basis of reconstructed data. We proposed a method based on a mixture of a Cox proportional hazards model and a binomial regression model. When applied to an analysis of condom use by female sex workers in India, the method yielded results quite similar to those obtained with a GEE-based approach in districts where initial condom use was low, but yielded results more plausible than those of the GEE-approach in districts where initial condom use was high.

## Appendix A

### Proof that the model can be fitted using standard binomial and Cox regression software

#### Independent data

Suppose that the data are collected at calendar time τ. At that time, we observe *n* independent FSWs. Let the observed data be $\left(D_{i},{\tilde{C}}_{i},{\tilde{F}}_{i},\delta_{i}\right)$, *i* = 1, …, *n*, where *D*_*i*_ is the calendar time at which FSW *i* started her career, *F*_*i*_ is the total duration of the career of woman *i* as a FSW, *C*_*i*_ is the amount of time during which woman *i* worked as a FSW before starting to consistently use the condom, ${\tilde{F}}_{i}=\tau -D_{i}$, ${\tilde{C}}_{i}=\min\left(C_{i},{\tilde{F}}_{i}\right)$, and *δ*_*i*_ = 1 if ${\tilde{C}}_{i}<{\tilde{F}}_{i}$ and 0 otherwise. Note that to be included in the study, woman *i* must be a FSW at time τ, i.e., *D*_*i*_ < *τ* and *D*_*i*_ + *F*_*i*_ > *τ*. Let *Y*_*i*_ = 1 if ${\tilde{C}}_{i}=0$ and *Y*_*i*_ = 0 otherwise. Then for inference about the distribution of *C*_*i*_ and *F*_*i*_, the likelihood function will be proportional to (with the slight abuse of notation whereby “Pr” may denote a probability or a density).

$$\begin{array}{lll} L= & \prod_{i=1}^{n} & \mathrm{Pr}\left[\left(\left({\tilde{F}}_{i},{\tilde{C}}_{i},\delta_{i}\right)\right|D_{i},D_{i}+F_{i}>\tau ,D_{i}<\tau \right]\\ & = & \prod_{i=1}^{n}{\left\{\mathrm{Pr}\left[\left(\left({\tilde{F}}_{i},{\tilde{C}}_{i},\delta_{i}\right)\right|D_{i},D_{i}+F_{i}>\tau ,D_{i}<\tau \right]\right\}}^{\delta_{i}}\\ & & {\left\{\mathrm{Pr}\left[\left(\left({\tilde{F}}_{i},{\tilde{C}}_{i},\delta_{i}\right)\right|D_{i},D_{i}+F_{i}>\tau ,D_{i}<\tau \right]\right\}}^{1-\delta_{i}} \end{array}$$

If we assume that *D*_*i*_, *C*_*i*_ and *F*_*i*_ are independent, we can factor the above probabilities as:

$$\begin{array}{ll} L & =\prod_{i=1}^{n}{\left\{\frac{S_{F}\left({\tilde{F}}_{i}\right)f_{C}\left({\tilde{C}}_{i}\right)}{\mathrm{Pr}\left[D_{i}+F_{i}>\tau |D_{i}\right]}\right\}}^{\delta_{i}}{\left\{\frac{S_{F}\left({\tilde{F}}_{i}\right)S_{C}\left({\tilde{C}}_{i}\right)}{\mathrm{Pr}\left[D_{i}+F_{i}>\tau |D_{i}\right]}\right\}}^{1-\delta_{i}}\\ & =\underset{\left(*\right)}{\underset{︸}{\left[\prod_{i=1}^{n}f_{C}{\left({\tilde{C}}_{i}\right)}^{\delta_{i}}S_{C}{\left({\tilde{C}}_{i}\right)}^{1-\delta_{i}}\right]}}\underset{\left(**\right)}{\underset{︸}{\left[\prod_{i=1}^{n}\frac{S_{F}\left({\tilde{F}}_{i}\right)}{\mathrm{Pr}\left[D_{i}+F_{i}>\tau \right]}\right]}} \end{array}$$

where *f*_*C*__,_*S*_*C*__,_ and *S*_*F*_ respectively denote the density of *C*_*i*_, the survival function of *C*_*i*_ and the survival function of *F*_*i*_ . We see that (*) is the likelihood for inference on the distribution of *C*_*i*_ based on a right-censored sample and that (**) is the likelihood for inference on the distribution of *F*_*i*_ on the basis of a sample of length biased data. Most importantly, we notice that (**) does not depend on the distribution of *C*_*i*_ and therefore inference about this latter distribution using only (*) will be valid and efficient.

Let us now rewrite (*) under the binomial regression with link function *g*() and Cox regression models given by Eq. 1 and Eq. 2, where this time *x*_*i*_ and *z*_*i*_(*t*) can be covariate vectors:

$$\begin{array}{l} L\left(\beta ,\gamma \right)=\underset{\left(a\right)}{\underset{︸}{\left[\prod_{i:Y_{i}=1}g^{-1}\left(\beta 'x_{i}\right)\right]}}\\ \;\times \underset{\left(b\right)}{\underset{︸}{\left[\prod_{i:Y_{i}=0,\delta_{i}=1}\left\{1-g^{-1}\left(\beta 'x_{i}\right)\right\}h_{0}\left({\tilde{C}}_{i}\right)e^{\gamma 'z_{i}\left({\tilde{C}}_{i}\right)}\exp\left(-\int_{0}^{{\tilde{C}}_{i}}h_{0}\left(t\right)e^{\gamma 'z_{i}\left(t\right)}\mathrm{dt}\right)\right]}}\\ \;\times \underset{\left(c\right)}{\underset{︸}{\left[\prod_{i:Y_{i}=0,\delta_{i}=0}\left\{1-g^{-1}\left(\beta 'x_{i}\right)\right\}\exp\left(-\int_{0}^{{\tilde{C}}_{i}}h_{0}\left(t\right)e^{\gamma 'z_{i}\left(t\right)}\mathrm{dt}\right)\right]}} \end{array}$$

Term (a) is the contribution to the likelihood of FSWs who consistently use the condom at the start of their career, term (b) is the contribution of FSWs who are observed to start CCU after the beginning of their career and term (c) is the contribution of FSWs who have not yet begun CCU at the time of data collection. By regrouping the terms, we can rewrite *L*(*β*, *γ*) as

$$\begin{array}{l} L\left(\beta ,\gamma \right)=\underset{\left(\#\right)}{\underset{︸}{\left[{\prod_{i=1}^{n}\left\{g^{-1}\left(\beta 'x_{i}\right)\right\}}^{Y_{i}}{\left\{1-g^{-1}\left(\beta 'x_{i}\right)\right\}}^{1-Y_{i}}\right]}}\\ \;\times \underset{\left(\#\#\right)}{\underset{︸}{\left[\prod_{i:Y_{i}=0}{\left\{h_{0}\left({\tilde{C}}_{i}\right)e^{\gamma 'z_{i}\left({\tilde{C}}_{i}\right)}\right\}}^{\delta_{i}}\exp\left(-\int_{0}^{{\tilde{C}}_{i}}h_{0}\left(t\right)e^{\gamma 'z_{i}\left(t\right)}\mathrm{dt}\right)\right]}} \end{array}$$

We recognize (#) as the likelihood for ordinary logistic regression based on the entire sample. As for (##), it is the full likelihood for inference on the parameters of the survival model for *C*_*i*_ with right-censored data and a time-varying covariate based on the sample comprised of FSWs who do not use the condom at the start of their career. Efficient semi-parametric inference about *γ* can be obtained by replacing (##) with the Cox partial likelihood, which is what most standard survival analysis software do. We therefore get that the overall likelihood is the product of the likelihood for ordinary binomial regression and the likelihood for Cox regression.

##### Clustered data

Let us assume that the data come from *K* clusters (e.g., districts). Standard software that implement binomial and Cox regression for clustered data will output consistent estimators of *β* and *γ*, along with their respective robust “sandwich” variance estimators. Typically, *β* is obtained by solving for *β* an estimating equation of the form $U\left(\beta \right)=\sum_{k=1}^{K}U_{k}\left(\beta \right)=0$ and *γ* is obtained by solving for *γ* an estimating equation of the form $V\left(\gamma \right)=\sum_{k=1}^{K}V_{k}\left(\gamma \right)=0$. As for the variance estimators, they are usually given by $\hat{V}\left(\hat{\beta }\right)={\hat{A}}_{\beta }{\hat{B}}_{\beta }{\hat{A}}_{\beta }$ with ${\hat{A}}_{\beta }=K^{-1}{\sum_{k=1}^{K}\left(\frac{\partial }{\partial \beta }U_{k}\left(\beta \right)\right|}_{\beta =\hat{\beta }}$ and ${\hat{B}}_{\beta }$ an empirical estimate of $B_{\beta }=E\left[\sum_{k=1}^{K}U_{k}\left(\beta \right)U_{k}\left(\beta \right)'\right]$, and mutatis-mutandis for the variance of $\hat{\gamma }$. Now if we put *θ* = (*β* ', *γ* ') ', then estimating *β* and *γ* separately amounts to solving $W\left(\theta \right)=\left(\begin{array}{c} U\left(\beta \right)\\ V\left(\gamma \right) \end{array}\right)=0$. It is easy to see that in this case, ${\hat{A}}_{\theta }=\left(\begin{array}{cc} {\hat{A}}_{\beta } & 0\\ 0 & {\hat{A}}_{\gamma } \end{array}\right)$ and $B_{\theta }=\left(\begin{array}{cc} B_{\beta } & B_{\beta \gamma }\\ B_{\gamma \beta } & B_{\gamma } \end{array}\right)$. But as a referee pointed out to us in a review of a previous version of this manuscript, the terms $B_{\beta \gamma }$ and $B_{\gamma \beta }$ are equal to zero. Thus a robust variance estimator of θ^=β^',γ^' is given by $\hat{V}\left(\hat{\theta }\right)=\left(\begin{array}{cc} {\hat{A}}_{\beta }{\hat{B}}_{\beta }{\hat{A}}_{\beta } & 0\\ 0 & {\hat{A}}_{\gamma }{\hat{B}}_{\gamma }{\hat{A}}_{\gamma } \end{array}\right)$. The practical implication of this result in our case is that the test on two degrees of freedom described earlier can be carried out by computing $Q=\frac{{\hat{\beta }}^{2}}{\hat{V}\left(\hat{\beta }\right)}+\frac{{\hat{\gamma }}^{2}}{\hat{V}\left(\hat{\gamma }\right)}$, which should follow a chi-squared distribution with two degrees of freedom under the null hypothesis of no intervention effect.

## Competing interests

The authors declare that they have no competing interests.

## Authors’ contributions

TD and BA surveyed the statistical literature and investigated the theoretical properties of the Cox-binomial method. TD ran the simulation study and re-analyzed the Indian data. TD wrote the manuscript. CML designed the estimation of retrospective data in absence of baseline and participated in the interpretation of the results. MA participated in the literature review, the development of the Cox-binomial method and the interpretation of the results. All authors read, critically reviewed and approved the final manuscript.

## Pre-publication history

The pre-publication history for this paper can be accessed here:

http://www.biomedcentral.com/1471-2288/14/2/prepub

## Supplementary Material

Additional file 1 **Table with additional analysis results.** This is a PDF file that contains a table that summarizes the results of the analysis of the Indian data with a single Cox model and where FSWs who start CCU at the beginning of their career are given an event time equal to 1 day. Click here for file

Additional file 2 **R code for the Cox-binomial method.** This is a PDF file from which the R code to implement the Cox-binomial analysis method described in this paper can be copied and pasted into R or into an R program editor. Click here for file
