# Supplementary material for: Assessing outcomes of large-scale public health interventions in the absence of baseline data using a mixture of Cox and binomial regressions
Source: BMC Med Res Methodol. 2014 Jan 7;14:2. doi: 10.1186/1471-2288-14-2 (PMC4029466; doi:10.1186/1471-2288-14-2)
Supplement: Additional file 1 — Table with additional analysis results. This is a PDF file that contains a table that summarizes the results of the analysis of the Indian data with a single Cox model and where FSWs who start CCU at the beginning of their career are given an event time equal to 1 day. [file 1471-2288-14-2-S1.pdf]

## Results obtained when fitting the Cox model to all individuals, with FSWs starting CCU at beginning of career given a time of CCU of 1 day = 1/365 year

| District | $n_{Cox}$ | $\hat{h}_1$ | $\hat{h}_2$ | $p_{Cox}$ |
|---|---|---|---|---|
| Belgaum | 397 | 0.230 | 0.431 | <0.001 |
| Bellary | 398 | 0.160 | 0.411 | <0.001 |
| Chennai | 349 | 0.133 | 0.503 | <0.001 |
| Chitoor | 395 | 0.022 | 0.132 | <0.001 |
| Coimbatore | 325 | 0.015 | 0.160 | <0.001 |
| Dharmapuri | 387 | 0.040 | 0.331 | <0.001 |
| East Godavari | 392 | 0.125 | 0.364 | <0.001 |
| Guntur | 386 | 0.040 | 0.378 | <0.001 |
| Madurai | 319 | 0.084 | 0.298 | <0.001 |
| Mumbai BB | 379 | 0.390 | 0.232 | 0.541 |
| Mumbai NBB | 354 | 0.368 | 0.212 | 0.023 |
| Mysore | 420 | 0.083 | 0.242 | <0.001 |
| Prakasam | 402 | 0.013 | 0.139 | <0.001 |
| Pune BB | 399 | 0.661 | 0.642 | <0.001 |
| Pune NBB | 251 | 0.531 | 0.505 | <0.001 |
| Salem | 319 | 0.080 | 0.374 | <0.001 |
| Shimoga | 338 | 0.122 | 0.372 | <0.001 |
| Thane BB | 397 | 0.813 | 0.862 | 0.145 |
| Thane NBB | 377 | 0.675 | 0.767 | <0.001 |
| Visakhapatnam | 405 | 0.058 | 0.420 | <0.001 |
| Yevatmal | 148 | 0.271 | 0.683 | <0.001 |
