# Supplementary material for: Assessing outcomes of large-scale public health interventions in the absence of baseline data using a mixture of Cox and binomial regressions
Source: BMC Med Res Methodol. 2014 Jan 7;14:2. doi: 10.1186/1471-2288-14-2 (PMC4029466; doi:10.1186/1471-2288-14-2)
Supplement: Additional file 2 — R code for the Cox-binomial method. This is a PDF file from which the R code to implement the Cox-binomial analysis method described in this paper can be copied and pasted into R or into an R program editor. [file 1471-2288-14-2-S2.pdf]

```
# This function performs Cox-logit analysis to test whether the
# rate of condom use at start of FSW career is the
# same pre- and post-intervention. Uses Sim data structure
#
# INPUTS:
# Donnees: A matrix with the data.
#       column 1 -> birthday in years (for instance July 1, 1972 would be 1972.5
#             and January 1, 1982 would be 1982.0)
#       column 2 -> time from birth until first of end of careeer as FSW or end of follow-up
#       column 3 -> date (in years) of beginning of career as FSW
#       column 4 -> date (in years) of beginning of consistent condom use
# Interv: date (in years) of intervention, i.e., time t0
# Analyses: date (in years) of data collection, i.e., time tau
# Link: link function for the logistic regression
#
# OUTPUT:
# This function returns a vector with the following elements:
#  "n.logit" -> number of FSW in the logistic regression part of the analysis
#  "n.cox" -> number of FSW in the Cox regression part of the analysis
#  "rate.pre" -> rate of acquisition of condom use before t0, i.e., h1
#  "rate.post" -> rate of acquisition of condom use after t0, i.e., h2
#  "prev.pre" -> prevalence of condom use at start of career before t0, i.e., pi1
#  "prev.post" -> prevalence of condom use at start of career after t0, i.e., pi2
#  "p.cox" -> p-value for the 2-sided test that coefficient in Cox model is 0
#  "p.logit" -> p-value for the 2-sided test that coefficient in logit model is 0
```

```
#  "p.2dl" -> p-value of the test on 2 degrees of freedom of the two hypotheses above combined


coxcutS <- function(Donnees,Interv=2004.0,Analyse=2009.0,Link="logit"){
  debut.FSW <- Donnees[,3]
  debut.condom <- Donnees[,4]
  fin.FU <- Donnees[,1]+Donnees[,2]
  # women who are FSW at time of data collection
  M <- length(debut.FSW)
  in.study <- (1:M)*((debut.FSW<Analyse)*(fin.FU>=Analyse))
  Donnees <- Donnees[in.study,]
  debut.FSW <- Donnees[,3]
  debut.condom <- Donnees[,4]

  # LOGIT analysis
  # y=1 if condom at beginning of career, 0 otherwise
  # x=1 if career started after t0, 0 otherwise
  y <- 1*(debut.condom==debut.FSW)
  x <- 1*(debut.FSW >= Interv)
  users.pre <- sum(y[x==0])
  users.post <- sum(y[x==1])
  model.logit <- glm(y~x,family=binomial(link=Link))
  p.val.logit <- 1-pchisq((model.logit$null.deviance-model.logit$deviance),1)
  n.logit <- length(y)
  n.pre <- length(y[x==0])
  n.post <- length(y[x==1])
  prev.pre <- users.pre/n.pre
```

```
prev.post <- users.post/n.post


# Cox analysis
# We only keep FSW with Y=0
in.cox <- (1:n.logit)[y==0]
Donnees <- Donnees[in.cox,]
debut.FSW <- Donnees[,3]
debut.condom <- Donnees[,4]
n.cox <- length(debut.FSW)
donnees.cox <- t(rep(0,4))
for(i in 1:n.cox){
  # case (a): career starts after t0, condom use starts after tau
  if((debut.FSW[i]>=Interv)&(debut.condom[i]>=Analyse)){
    t1 <- 0
    t2 <- Analyse-debut.FSW[i]
    d <- 0
    z <- 1
    donnees.cox <- rbind(donnees.cox,t(c(t1,t2,d,z)))
  }
  # case (b): career starts after t0, condom use starts before tau
  if((debut.FSW[i]>=Interv)&(debut.condom[i]<Analyse)){
    t1 <- 0
    t2 <- debut.condom[i]-debut.FSW[i]
    d <- 1
    z <- 1
    donnees.cox <- rbind(donnees.cox,t(c(t1,t2,d,z)))
```

```
}

# case (c): career starts before t0, condom use starts after tau

if((debut.FSW[i]<Interv)&(debut.condom[i]>=Analyse)){

  t1 <- 0

  t2 <- Interv-debut.FSW[i]

  d <- 0

  z <- 0

  donnees.cox <- rbind(donnees.cox,t(c(t1,t2,d,z)))

  t1 <- Interv-debut.FSW[i]

  t2 <- Analyse-debut.FSW[i]

  d <- 0

  z <- 1

  donnees.cox <- rbind(donnees.cox,t(c(t1,t2,d,z)))

}

# case (d): career starts before t0, condom use starts after t0 but before tau

if((debut.FSW[i]<Interv)&(debut.condom[i]<Analyse)&(debut.condom[i]>=Interv)){

  t1 <- 0

  t2 <- Interv-debut.FSW[i]

  d <- 0

  z <- 0

  donnees.cox <- rbind(donnees.cox,t(c(t1,t2,d,z)))

  t1 <- Interv-debut.FSW[i]

  t2 <- debut.condom[i]-debut.FSW[i]

  d <- 1

  z <- 1

  donnees.cox <- rbind(donnees.cox,t(c(t1,t2,d,z)))
```

```
  }
  # case (e): career and condom use both start before t0
  if((debut.FSW[i]<Interv)&(debut.condom[i]<Interv)){
    t1 <- 0
    t2 <- debut.condom[i]-debut.FSW[i]
    d <- 1
    z <- 0
    donnees.cox <- rbind(donnees.cox,t(c(t1,t2,d,z)))
  }
}
donnees.cox <- donnees.cox[-1,]
t1 <- donnees.cox[,1]
t2 <- donnees.cox[,2]
d <- donnees.cox[,3]
z <- donnees.cox[,4]
library(survival)
model.cox <- coxph(Surv(t1,t2,d)~z)
p.val.cox <- 1-pchisq(2*(model.cox$loglik[2]-model.cox$loglik[1]),1)
donnees.cox.pre <- donnees.cox[donnees.cox[,4]==0,]
donnees.cox.post <- donnees.cox[donnees.cox[,4]==1,]
personyears.pre <- sum(donnees.cox.pre[,2]-donnees.cox.pre[,1])
personyears.post <- sum(donnees.cox.post[,2]-donnees.cox.post[,1])
cases.pre <- sum(donnees.cox.pre[,3])
cases.post <- sum(donnees.cox.post[,3])
taux.pre <- cases.pre/personyears.pre
taux.post <- cases.post/personyears.post
```

```
  # hypothesis test on 2 degrees of freedom
  null.loglik <- model.cox$loglik[1]-model.logit$null.deviance/2
  h1.loglik <- model.cox$loglik[2]-model.logit$deviance/2
  lrt.stat <- 2*(h1.loglik-null.loglik)
  p.val.2dl <- 1-pchisq(lrt.stat,2)

  # preparation of the output
  output <- c(n.logit,n.cox,taux.pre,taux.post,
        prev.pre,prev.post,p.val.cox,p.val.logit,p.val.2dl)
  names(output) <- c("n.logit","n.cox","rate.pre","rate.post",
          "prev.pre","prev.post","p.cox","p.logit","p.2df")
  return(output)
}
```
